# A noninvasive eDNA tool for detecting sea lamprey larvae in river sediments: Analytical validation and field testing in a low‐abundance ecosystem

**DOI:** 10.1111/jfb.15056

**Published:** 2022-04-19

**Authors:** Miguel Baltazar‐Soares, Adrian C. Pinder, Andrew J. Harrison, Will Oliver, Jessica Picken, J. Robert Britton, Demetra Andreou

**Affiliations:** ^1^ Department of Life and Environmental Sciences Faculty of Science and Technology, Bournemouth University Dorset UK; ^2^ MARE – Marine and Environmental Sciences Centre ISPA – Instituto Universitário Lisbon Portugal; ^3^ Jacobs Southampton UK; ^4^ The Centre for Environment, Fisheries & Aquaculture Science, Lowestoft Laboratory Lowestoft UK; ^5^ School of Biological and Chemical Sciences Queen Mary University of London London UK; ^6^ Game & Wildlife Conservation Trust Salmon and Trout Research Centre Wareham UK

**Keywords:** biological conservation, environmental DNA, freshwater ecology, monitoring, sea lamprey

## Abstract

Anthropogenic activities are increasingly threatening aquatic biodiversity, especially anadromous species. Monitoring and conservation measures are thus required to protect, maintain and restore imperilled populations. While many species can be surveyed using traditional capture and visual census techniques, species that use riverine habitats in a less conspicuous manner, such as sea lamprey *Petromyzon marinus*, can be more challenging to monitor. Sea lamprey larvae (ammocoetes) can spend several years in freshwater burrowed within soft sediments, inhibiting their detection and assessment. Here, we present a qPCR assay based on the detection of environmental DNA (eDNA) to identify the presence of ammocoetes burrowed in the sediment. We present an extensively validated method that ensured both species‐specificity of the assay as well as the capacity to detect ammocoetes when abundances are low. Experiments on burrowing activity suggested that most of the DNA released into the sediment occurs during burrowing. Overall, we demonstrate this new molecular‐based tool is an efficient and effective complement to traditional monitoring activities targeting larval stages of sea lampreys.

**SIGNIFICANCE STATEMENT**

With freshwater biodiversity becoming threatened worldwide by human activities, monitoring the population dynamics of fluvial species is critical. Here we introduce a different take on the application of environmental DNA to river monitoring. With the objective of creating a molecular tool to screen sedimentation zones, we developed and validated an eDNA assay that, from the sediment, is able to detect the presence of sea lamprey ammocoetes – under low abundances – burrowed in riverbeds.

## 1 INTRODUCTION

Anthropogenic activities in freshwater ecosystems can result in deleterious effects on biodiversity (Dodds *et al*., 2013), including on those species with complex life cycles, such as anadromous fishes (Dias *et al*., 2017). Effective conservation management of freshwater biodiversity is reliant on robust monitoring that enables long‐term spatial and temporal patterns to be detected (Radinger *et al*., 2019). This monitoring can, however, be challenging in many freshwater ecosystems, with biases in sampling methods leading to issues associated with false‐negative data, especially if relying on visual detection and counting (Thomsen *et al*., 2012). Threatened species that have complex life cycles, and with life stages that occupy a range of different habitats, can then present further challenges to sampling efficacy within monitoring programmes (Radinger *et al*., 2019).

An alternative to the application of capture and visual sampling methods is the use of environmental DNA (eDNA) methods that are designed to detect the DNA of organisms within environmental samples, such as water (Thomsen & Willerslev, 2015). Although eDNA‐based detection methods require extensive validation prior to their application in the field, their capacity to detect DNA from environmental samples without capturing or visually confirming the species presence is becoming increasingly cost‐efficient (Doi *et al*., 2017; Evans *et al*., 2017; Ficetola *et al*., 2008). Correspondingly, the application of eDNA detection to monitoring biodiversity is now well established, with a broad range of applications, including species detection in either an ancient or contemporary context, from the reconstruction of past faunal or floral assemblages to the identification of range expansions/biological invasions (Bohmann *et al*., 2014). In the freshwater environment, it has been used to document habitat utilization, detect the presence of rare and invasive species, and quantify spawning activity in predefined areas (Bracken *et al*., 2019; Stoeckle *et al*., 2017; Thomsen & Willerslev, 2015), including the extent of upstream spawning migrations in anadromous fish species (Antognazza *et al*., 2019).

The sea lamprey *Petromyzon marinus* is a jawless vertebrate, one of the few surviving species of this ancient group of animals (Guo *et al*., 2017). Its complex life cycle comprises stages in fresh‐, brackish and saltwater. Mating occurs in fresh water in primitive nests built by males to attract females (Guo *et al*., 2017). Batches of eggs are deposited within the gravel substrate, where they reside for an extended larval phase (~5–6 years) until they emerge during the ontogenetic metamorphosis from larvae (ammocoetes) to young juveniles (transformers), which then drift downstream to deeper areas of low water velocity before settling into areas of soft sediment (Pinder *et al*., 2016). Their downstream migration to sea is accomplished by successive settlement/emergence events; a process lasting at least 5 years (Quintella *et al*., 2005). In the sea, adults are parasitic on fish until sexual maturity, when they then return to freshwater (Sorensen & Vrieze, 2003).

In Europe, threats to the conservation status of *P. marinus* include river fragmentation, habitat loss and declining water quality (Pinder *et al*., 2016), resulting in protected status through designation under Annex II of the EU Habitats Directive (Directive 92/43/EEC). Robust population monitoring is required to ensure that populations achieve the regulatory target of ‘favourable conservation status’, with monitoring strategies usually targeting the freshwater stages, especially nest counts during spawning activity or abundance estimates of ammocoetes in the sediment (Guo *et al*., 2017; Mateus *et al*., 2012). As ammocoetes remain in fresh water for several years, targeting sediment sites to infer ammocoete presence and/or abundance could provide evidence for the sustainability of sea lamprey populations within specific rivers and habitats. Correspondingly, the aim here was to develop a qPCR assay designed and tested to amplify sea lamprey DNA from sediment samples, to provide a conservation monitoring tool with the capability of being utilized all year round.

## 2 MATERIALS AND METHODS

### 2.1 Sampling site and identification of sediment deposition sites

This work was performed on the River Frome, Southern England, where adults spawn in the lower reaches, but with considerable interannual variation in numbers (Pinder *et al*., 2016), across two field campaigns: May to July of 2017 and 2018. Because locations of burrowing sites in the River Frome were largely unknown, the first field campaign aimed to identify areas where sea lamprey ammocoetes burrowed. Here we targeted river meanders with sediment deposition, known to be ideal for ammocoete burrowing (Quintella *et al*., 2007).

**FIGURE 1:**
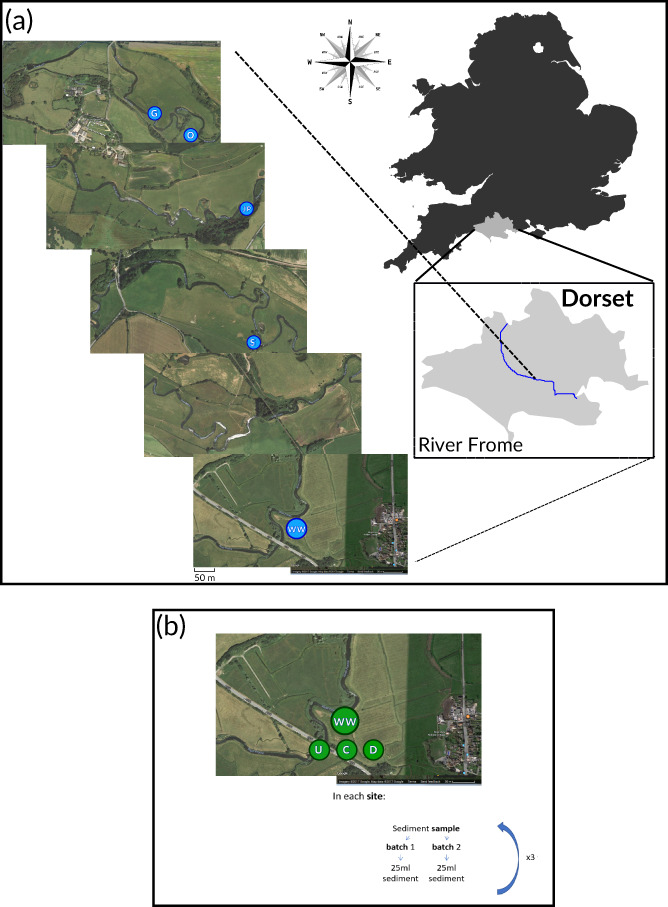
Sampling sites and strategy for environmental DNA screens. (a) Riverside sites of sediment sampling along a transect of the river Frome. GPS coordinates for the sampled sites: G, 50.6789, −2.1794; O, 50.6780, −2.1767; JP, 50.6756, −2.1607; S, 50.6764, −2.1457; WW, 50.6778, −2.1157. (b) Sampling strategy adopted for the monitoring *in situ* strategy at the five sampling sites. U, location upstream of the site; C, location at the site; D, location downstream of the site. Sediment was extracted six times for each location within the sites

Sites where sea lamprey ammocoetes were positively identified were then visited again in 2018, where sampling was repeated to reaccess presence and collect eDNA samples. Thus, in the second field campaign, we focused on five sites (with the most distant being 5 km apart) along a 13 km stretch of the lower River Frome (Figure 1a). Sampling activity duration, accounted for approximately 14 weeks in total.

### 2.2 Identification of sea lamprey ammocoetes *via* mitochondrial DNA barcoding

With the nonmigratory brook lamprey *Lampetra planeri* being prevalent throughout the river system, a primary requirement was validation of the ability to distinguish between brook and sea lamprey. Ammocoetes of sea and brook lamprey are virtually indistinguishable prior to the onset of pigmentation, but thereafter it has been proposed that morphological discrimination can be based on pigmentation around the oral hood and on the caudal fin, where it is present in sea lamprey but not in brook lamprey (Kelly & King, 2001).

To ensure that pigmentation was a species‐defining trait, six lamprey ammocoetes were extracted from the sediment using a dipnet (50 cm diameter), euthanised (anaesthetic overdose, tricaine methanesulfonate) and preserved (99% ethanol). The six ammocoetes comprised three pigmented (putative sea lamprey) and three nonpigmented (putative brook lamprey) specimens. Species identification was verified with mitochondrial DNA (mtDNA) barcodes. Extraction of DNA was performed with a commercially available extraction kit DNeasy Blood and Tissue Kit (Qiagen, Hilden, Germany) and followed the manufacturer protocol. Amplification targeted a portion of the control region in the mitochondrial DNA using a mix of primers from two different studies, LampFor5′‐ACACCCAGAAACA GCAACAAA‐3′ LampRev5′‐GCTGGTTTACAAGACCAGTGC‐3′, CR1 (5′‐ACAACACCAGCTACCCCC‐30) and CR5 (50‐CCTAAGGGGGTT‐GACGGC‐3′), that were specifically designed for brook and sea lamprey (Almada *et al*., 2008; Rodríguez‐Muñoz *et al*., 2004). Amplification was through polymerase chain reaction (PCR) under the following conditions: 95°C for 15 min for *Taq* polymerase activation, followed by 35 cycles of a denaturation step at 94°C for 30 s, an annealing step at 55°C for 30 s and an elongation step at 72°C for 30 s, and a final elongation step at 72°C for 10 min. The PCR mix was composed of 5 μl of Qiagen MasterMix, 0.4 μl of forward and reverse primer (5 μM), 2.6 μl of purified water and 2 μl of DNA (~100 ng μl^−1^). To confirm successful amplification, electrophoresis was carried out at 70 V for 45 min in 1% agarose gels stained with SYBR‐green. Forward sequencing was outsourced to GENEWIZ; sequences were aligned, curated manually in BioEdit (Hall, 1999) and compared against those deposited in the National Center for Biotechnology Information (NCBI) database with the *megablast* algorithm (McGinnis & Madden, 2004). To statistically assess the clustering of the DNA originating from individuals with pigmented tails with known sea lamprey sequences, we constructed a neighbour‐joining phylogenetic tree (1000 bootstraps) in MEGA v6 (Tamura *et al*., 2013).

### 2.3 Design of a quantitative PCR assay to detect environmental DNA: Species‐specific primers and probe

Due to the high taxonomic representation of sea lamprey mtDNA in the NCBI database, we chose to design primers in conserved regions of the sea lamprey mitochondrial molecule. We first verified the phylogenetic relationship among cyclostomes that closely relate to sea lamprey and whose sequences were available in the NCBI. We focused on mtATP8 and mtATP6, which are contiguous in the mtDNA genome, because a large number of brook and river lamprey sequences, *i.e*., the closely related taxa whose distribution largely overlaps that of sea lamprey's freshwater phase, were available. From there on, we built a database using only sea lamprey, brook lamprey, river lamprey (*Lampetra fluviatilis*) and other lamprey as outgroups, which comprised 100 sequences (36 *L. fluviatilis*, 19 *L. planeri*, 38 *Lampetra* spp., two *Ichthyomyzon gagei*, three *Ichthyomyzon unicuspis*, one *Ichthyomyzon fossor*, one *Lampetra appendix*, one *Lampetra aeyptera*) aligned against two sea lamprey mitochondrial genomes. The objective was to identify mitogenomic areas that for the target species *P. marinus* would exhibit high levels of intraspecific conservatism and, concomitantly, would ensure that high interspecific diversity was present, as this balance decreases the risk of cross‐species amplification. Primer/probe design was conducted in Primer3 on the chosen mitochondrial gene (Untergasser et al., 2012). Phylogenetic relationships across cyclostomes and primer alignment with reference sequences are shown in Supporting Information Figure S1.

Prime and probe synthesis was outsourced to ThermoFisher Scientific. Testing the efficiency and specificity of the primers used a three‐stage process: (a) confirm assay sensitivity in detecting various concentrations of sea lamprey DNA in TE solution; (b) confirm primer specificity in amplifying only sea lamprey DNA; and (c) verify primer efficiency to detect DNA molecules extracted from environmental samples (including in the controlled presence of sea lamprey ammocoetes).

#### 2.3.1 1. Sensitivity to variable concentrations of sea lamprey DNA

The primers and probe were designed on the mitochondrial region, corresponding to genes ATP6–ATP8, which we found to be highly conserved between lamprey species. The highest discriminatory power for sea lamprey was found in the ATP8 region, as the probability of cross‐reactivity with closely related species was much lower.

The efficiency and sensitivity of the designed primer and probe were first tested against solutions of standardized DNA concentration to demonstrate that amplification occurs even under low DNA concentrations. Amplification and detection of probe fluorescence were tested at seven different DNA concentrations, obtained after 10‐fold serial dilutions to the lowest concentration of 5 ng μl^−1^ solution. Extracted DNA of adult sea lampreys was used to make dilutions (Baltazar‐Soares *et al*., unpublished data). The assay was synthesized at ThermoFisher, available with the ID APWCW7W, and consisted of a Taqman MGB probe (5‐CACCATCTCTACTAAACAAGTT‐3) labelled with the fluorescent dye FAM at the 5′ end and with a non‐fluorescent quencher MGBNFQ at the 3′ end and two primers, forward 5‐GATCCTGCCCCTTGATTCTCTA‐3 and reverse 5‐TCATGGTCAGGTTCAAGTGGAT‐3. All quantitative real‐time PCR (qRT‐PCR) reactions were conducted in 20 μl reactions: 10 μl of TaqMan Gene Expression Master Mix, 1 μl of assay mix and 2 μl of DNA template. Thermocycler conditions were the following: holding stage at 50°C for 2 min, initial denaturation at 95°C for 10 min followed by 40 cycles of denaturation at 95°C for 15 s and annealing at 60°C for 1 min in a StepOnePlus Real Time System. All reactions were performed in triplicate using filter tips, with negatives included in each 96‐well plate, *i.e*., a total of three negatives. Note that these sequential dilutions were later used to build standard calibration curves in all subsequent reactions.

Detection of amplified product was quantified with cycle Threshold thresholds with specialized ABI software. All laboratorial equipment was exposed to UV light for 20 min prior to performing any protocol. The mixture of reagents and preparation of plates was done in a sterilized hood (constant velocity) that was bleach‐cleaned for 2 h and left to dry overnight prior to work.

#### 2.3.2 2. Validation against cross‐species amplification

To eliminate false positives due to amplification in nontarget species, *in silico* testing of the primers and probe was carried out *via* NCBI Blast (Ye *et al*., 2012). In addition, to further ensure specificity, the 16 most common freshwater fishes in the River Frome, and surrounding waterbodies and neighbouring river catchments, were screened for amplification with the assay using 10 ng μl^−1^ DNA with the above conditions. Species tested were brook lamprey, roach (*Rutilus rutilus*), common bream (*Abramis brama*), chub (*Squalius cephalus*), minnow (*Phoxinus phoxinus*), perch (*Perca fluviatilis*), dace (*Leuciscus leuciscus*), bleak (*Alburnus alburnus*), grayling (*Thymallus thymallus*), brown trout (*Salmo trutta*), Atlantic salmon (*Salmo salar*), gudgeon (*Gobio gobio*), European eel (*Anguilla anguilla*), carp (*Cyprinus carpio*), European barbel (*Barbus barbus*) and shad (*Alosa* spp.).

#### 2.3.3 3. Efficiency in detecting sea lamprey DNAfrom sediment samples

The sensitivity of the assay to detect DNA in the sediment was then tested. We also inferred whether the time spent in a sample of sediment could influence the efficiency of the assay. Sampling was performed with a dipnet (3 m handle, 250 μm mesh size, 50 cm diameter) that was dipped into the sediment down to 2 m, brought to surface and then emptied into 4 l trays for ammocoete screening. This activity was performed on site JP (Figure 1), as this was identified the site with the highest likelihood of encountering sea lamprey ammocoetes in the field surveys of summer 2017. The sampling concluded when three ammocoetes had been captured that had been identified as sea lamprey by their pigmented caudal fin (a trait that was confirmed with molecular markers that discriminate sea lampreys ammocoetes; see Results). These ammocoetes were placed into individual falcon tubes (50 ml) that had been filled with 25 ml of sediment and approximately 20 ml of water collected from upstream locations above a weir that is largely impassable to sea lampreys and where their absence was confirmed by electric fishing during summer 2017 and 2018. Following the ammocoetes burying into the sediment, approximately 20 mg of sediment was removed at three time intervals (*t* + 5, *t* + 10 and *t* + 20 min). At the end of the process, the ammocoetes were released alive to their capture location. DNA was then extracted from all the collected sediment samples alongside a negative, which comprised a sample of pure sediment prior to insertion of the lamprey. Inhibition was reduced by using a commercially available kit.

DNA extraction from the sediment samples was performed with a commercially available extraction kit (DNAeasy PowerSoil Kit, Qiagen, Hilden, Germany) following the manufacturer's protocol.

#### 2.3.4 4. In‐field screening of sediment deposition sites

Five field sites were targeted as monitoring locations for detecting sea lamprey ammocoetes, with the areas selected being those where sea lamprey ammocoetes were found in the field campaign of the previous year. The objective was to infer the sensitivity of the qPCR assay to detect sea lamprey DNA outside the putatively optimal burrowing location. Sediments were collected with the 3 m handle and 50 cm diameter dipnet both upstream (U) and downstream (D) of the target sediment‐deposition location (C) at a depth of 2 m. Sediment was then deposited in a 4 L tray specific to each location U, C or D (to avoid cross‐site contamination) and the presence of ammocoetes was screened manually. Each location was sampled with the dipnet six times for replication, iterating the deposition of sediment in the tray and respective manual screen. Trays were rinsed after each sediment deposition. We collected a subsample of 25 ml of sediment from each of the six replicates. DNA was extracted from two randomly selected subsamples per U, C or D location. One site per day was sampled to allow field material to be disinfected from DNA molecules with bleach overnight.

The sampling scheme with the specific geographic location of each target sediment‐deposition site, plus the upstream and downstream collection points within the 13 km transect, are represented in Figure 1. The particle size of each sediment site was estimated to ensure that the sediment of C locations was composed of finer particles. Particle size was measured in a Mastersizer 3000 (Malvern Panalytical, Malvern, UK) *via* laser light scattering of three replicates per location site. Both the standards and each chosen sample were amplified three times.

### 2.4 Statistical analyses and graphical visualization

All statistical analyses were performed in R 3.4.1 (Team, 2013). To investigate the efficiency of the qPCR assay in relation to time in the sediment (as described in section 3), we performed a nested ANOVA with time points nested within specimens: CT_threshold_ ~ specimens/time point.

### 2.5 Ethical statement

The care and use of experimental animals complied with Environment Agency animal welfare laws, guidelines and policies as approved by permit reference EP/EW030‐E‐866/11921/01.

## 3 RESULTS

### 3.1 Species identification

**FIGURE 2:**
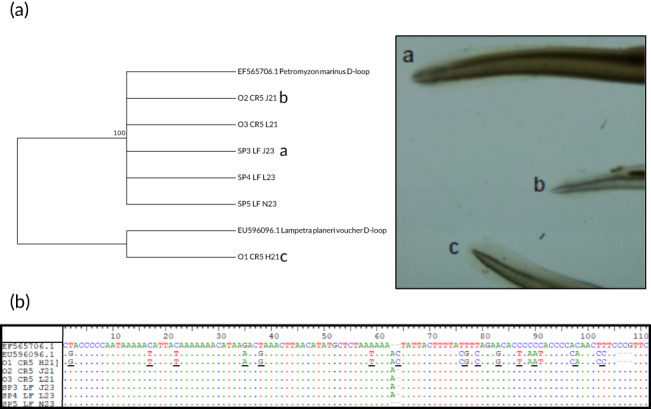
Relationship between morphological and molecular traits in the identification of sea lamprey ammocoetes. (a) Phylogenetic tree constructed with the neighbour‐joining method (Saitou & Nei, 1987). The percentage of replicate trees in which the associated taxa clustered together in the bootstrap test (1000 replicates) is shown next to the branches (Felsenstein, 1985). Letters a, b and c relate to the leftmost picture, under a microscope lens, of the respective pigmented and nonpigmented tails. (b) Partial representation of the D‐loop fragment used to barcode ammocoetes with morphologically distinct tail pigmentation. Species‐specific segregation sites are underlined

Sequencing of the six ammocoetes (three pigmented and three nonpigmented) produced a 419 base‐pair fragment and molecular inference of species‐specific polymorphisms. Phylogenetic relationship places pigmented tails with sea lampreys and nonpigmented with brook lamprey (Figure 2). BLAST results can be consulted, for all sequences, in Supporting Information Tables S1–S6. All sequences obtained in this work are deposited in GenBank (MW438278–MW438283).

### 3.2 Development and validation of the environmental DNA‐based tool

For the 10‐fold serial dilution of sea lamprey DNA, the limit of detection was 5 × 10^−5^ ng μl^−1^, with a mean cycle threshold value (*C*_t_) of 37 (SD ± 0.02). The *C*_t_ values with DNA dilutions in later cycles (>37), corresponding to 5 × 10^−6^ and 5 × 10^−7^ ng μl^−1^, were unreliable due to their probability of detection being below the 95% confidence level, and as such we used six standard concentrations throughout the qPCR assay. There was no cross‐amplification with nontarget species, and primer pair/probe blast analysis revealed the sea lamprey mitochondrial genome as first and only statistically robust hit for the forward primer, the reverse primer and the probe simultaneously (Supporting Information Tables S7–S9).

### 3.3 Effect of burrowing activity and time since burrowing on DNA detection

**FIGURE 3:**
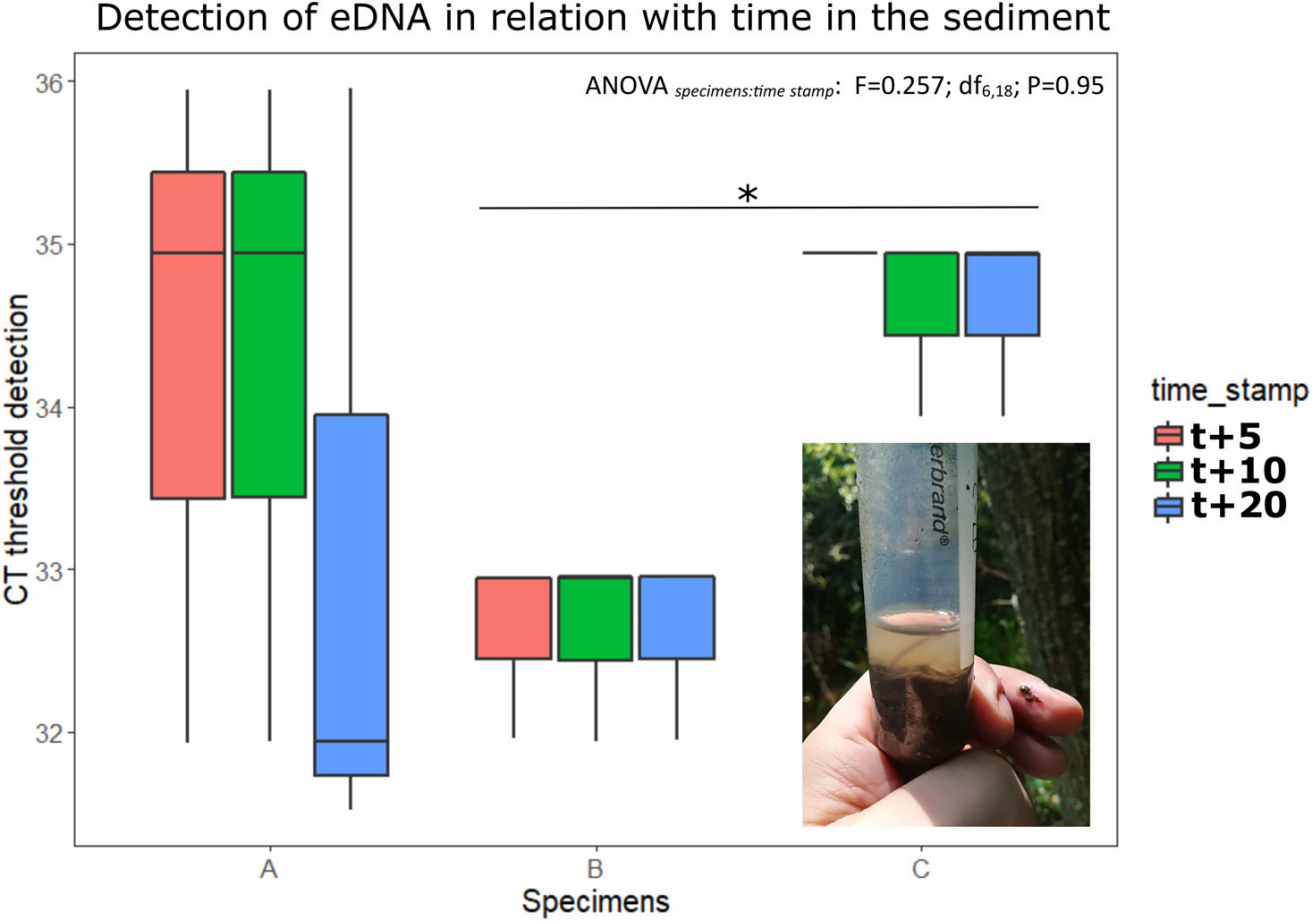
Detection of sea lamprey DNA after sediment exposure to live specimens. Assay sensitivity to duration of ammocoete burial. Significant differences in detection threshold only identified between individuals. CT, Cycle Threshold

Sea lamprey DNA was detected in sediment samples after exposure with live juveniles for the three time intervals, with *C*_t_ ranging from 31.93 to 35.95, although there were no significant differences in terms of *C*_t_ detection as a response to time in the sediment (ANOVA_specimens:time stamp_: *F*_6,18_ = 0.257, *P* = 0.95) (Figure 3). However, there was a significant difference between individual detection values.

### 3.4 Field application of qPCR assay to detect eDNA of ammocoetes in low abundance conditions

**FIGURE 4:**
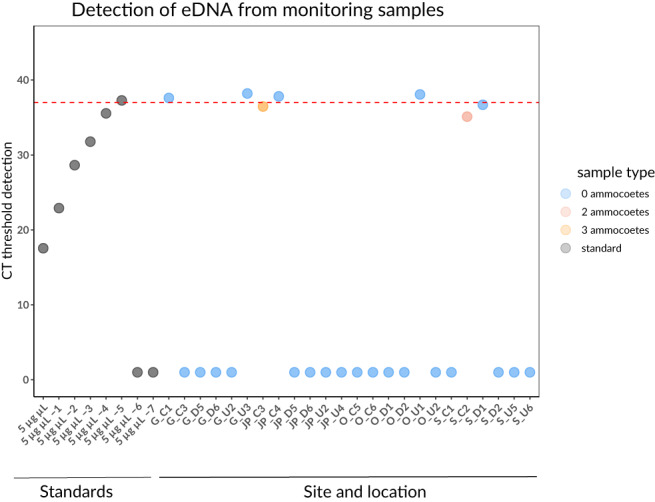
qPCR detection thresholds for field samples. Application of qPCR assay to detect environmental DNA molecules among sediment samples collected across river sites as well as standards. Line at *y*(*C*_t_) = 38 depicts the standardized detection threshold. Values with *C*_t_ = 0 represent no detection. CT, Cycle Threshold

Particles in sediment patches where sea lamprey ammocoetes were found had a mean size of 27.18 μm (±0.68 SD), and thus were smaller than those estimated from immediately upstream and downstream locations (*U*_average particle size_ = 38.72 μm, ±1.91 SD; *D*_average particle size_ = 38.72 μm, ±1.91 SD); ANOVA_site: location_: *F*_10,60_ = 184.68; *P* < 0.001; Supporting Information Figure S1). The application of the qPCR assay to the five river sites in summer 2018 was based on the sites where they were captured in 2017. However, despite intensive effort (comprising 3 weeks of daily monitoring in all possible sedimentation zones of the transect), only two ammocoetes were found at site S (S_C2) and three ammocoetes at site JP (JP_C). Application of the qPCR assay to those samples revealed *C*_t_ of 35.11 and 36.46, respectively. All other samples where no ammocoetes were found during sediment sampling, as well as field negatives, had no detectable sea lamprey DNA (with *C*_t_ values above the detection confidence threshold; Figure 4).

## 4 DISCUSSION

With short stays in rivers as adults to spawn but with extended development phases as larvae burrow in sediment, monitoring the presence of sea lampreys in freshwater environments is a challenging task. Indeed, when reproduction and nesting events are rare or unable to be detected due to unsuitable river conditions (such as turbidity or impossibility to access nesting sites), documenting the presence of this species in fresh waters must resort to trapping, dredging of sediment patches and/or electric fishing (Harvey *et al*., 2010). The advent of eDNA‐based detection tools came as a complement to traditional monitoring activities and, with this work, we show that ammocoete monitoring using eDNA is possible and its addition to the sea lamprey monitoring toolkit should provide considerable conservation benefits.

Our assay was shown to be highly efficient in detecting sea lamprey DNA in sediment samples. Indeed, it was able to detect the presence of sea lamprey larval DNA after a minimum exposure of only 5 min in 25 ml of sediment, with the time of exposure not inducing variation in the detection thresholds. This can be interpreted in light of their burrowing behaviours; after the initial burrowing activity, the ammocoete held still as soon as it got partially burrowed in the sediment, with stillness being characteristic of their daylight behaviour (Quintella *et al*., 2005). While the DNA detected was likely to have been shed during the intense period of burrowing activity, we acknowledge also that the confined environment of a 50 ml Falcon tube prevented the eDNA from being washed away.

Whilst it has been suggested that the concentration of extra‐organismal DNA in a given environment is a function of an individual's metabolic activity, behaviour and abundance (Lacoursière‐Roussel *et al*., 2016), we argue that the capacity to detect extra‐organismal DNA also varies with the proximity of the sampled patch to the presence of individuals. Interestingly, we further observed significant differences between individual *C*_t_ thresholds, suggesting that variation in individual DNA release (whether by movement, skin shedding or excretion) could also be a determining factor to consider in qPCR assays to detect eDNA, on top of others such as density of target organisms (Stoeckle, Beggel, *et al*., 2017). Sea lamprey eDNA was detected in field samples with extremely low ammocoete abundances (with a *C*_t_ range of 35.11–36.46). This observation suggests that ammocoete density in natural sediment patches should be considered as a factor influencing detection rates of molecular material. Notably, there was no detection of qPCR reaction inhibition, as all replicates exhibited positive amplification and all negatives were indeed negative, while positives were concomitantly tested. These results suggest that although negative results should be treated with some caution, given uncertainty around the degradation time of eDNA in the substrate (Thomsen & Willerslev, 2015) and the duration of the ammocoete presence in sediment beds (Quintella *et al*., 2005), these can be overcome with further work on understanding the persistence of DNA following the movement of a species to a different location. Recently, a qPCR assay using water samples was developed to identify key spawning areas for sea lamprey, including an investigation of the impact of physical barriers that prevent the species fully utilizing its potential spawning habitat (Bracken *et al*., 2019). Whilst useful for mapping the spatial distribution of spawning areas, the utility of the tool is limited by the short temporal window during which spawning occurs. Overall, our sediment‐based assay potentially complements the recent work of Bracken *et al*. (2019), as it allows the screening of sediment patches downstream of spawning grounds and thus estimates reproductive success. Because DNA molecules might persist for longer periods in sediment than in running water (Ostberg *et al*., 2019), this potentially increases the chances of false positives at higher population densities. Thus, these two methods should be considered as complementary in the dedicated monitoring of sea lampreys.

The qPCR assay developed here to detect sea lamprey eDNA will be of most benefit to ongoing ammocoete monitoring if used as a complement to other general management practices, such as quantitative quadrat‐based or semiquantitative electric fishing (Cowx, 2003). Furthermore, DNA‐based monitoring enables increased knowledge on sea lamprey ammocoete distribution to be generated, including identifying potential nursery habitats in terms of water depth. Due to methodological constrains to the application of electric fishing beyond certain depths, existing monitoring activities are only able to target sediment beds as deep as 1 m. However, recent research developments of sea lamprey ammocoete habitat utilization suggest preferences for deeper nurseries, *i.e*., >2 m (Pinder *et al*., 2016; Taverny *et al*., 2012). Through the analyses of sediment cores from deeper regions of a river, qPCR screening also has the potential to facilitate the assessment of this species’ downstream migration to the sea and investigate the accuracy of expected successive settlement/emergence events from one sediment patch to another. The qPCR assay here applied offers an improvement to sea lamprey current monitoring strategies. However, it is important to consider inhibition across spatial scales of sediment sampling, as the presence of inhibitors can be specific to sediment patches. As we were unable to do this for all patches, pervasive failure to detect sea lamprey DNA should be interpreted with caution, despite the extensive absence of ammocoetes in sediment patches. Alternatively, we suggest future work should include the use of the Applied Biosystems Environmental Master mix, which removes inhibitors from environmental samples, and/or an internal amplification control.

By targeting the sediment, we are proposing a holistic sea lamprey monitoring strategy able to be employed all year round to river areas that are critical for the species' life history. Notwithstanding, it is important to consider that eDNA is likely to persist in the sediment for longer periods than in running water and thus extensive sediment‐based monitoring should be especially careful with false positives (Nelson‐Chorney *et al*., 2019). Because this work was developed in a river with a low sea lamprey population density, the very low number of ammocoetes encountered not only inhibited the widening of investigations but also compromised the strength of field observations. The low availability of sea lamprey ammocoetes was thus a limiting factor here, and future studies should consider using a river of higher ammocoete abundances. This would permit the transfer of ammocoete into mesocosms without impacting the dynamics of the natural populations. The controlled conditions provided in mesocosms would then allow more accurate quantification of the effect of ammocoete density on their probability of detection, as well as allowing larger volumes of water to be used in experiments that test DNA decay rates and the effect of time since DNA deposition on detection probabilities. Also, the use of a river with higher sea lamprey ammocoete abundances should result in the number of occupied sediment patches being higher, thus providing more natural replicates to test the sensitivity of the assay across larger spatial scales in relation to both burrowing sites and the proximity to the river mouth, and to better manage potential sources of inhibition on large‐scale sediment samples.

## AUTHOR CONTRIBUTIONS

All authors contributed to the design of the experiment. M.B.S., J.P. and W.O. performed the field work and completed the analyses. M.B.S. led the writing of the manuscript. All authors contributed to revising the manuscript and all authors approved its submission.

## Supporting information


**Supporting Information Figure S1**Phylogenetic relationship based on the ATP8–ATP6 region. The quantitative PCR assay relied on primers designed to amplify only sea lamprey DNA. (A) Unrooted phylogenetic tree constructed with the neighbour‐joining method (Saitou & Nei, 1987) and utilizing 100 cyclostome sequences retrieved from the National Center for Biotechnology Information. Species utilized were the sea lamprey (*Petromyzon marinus*), Lampetra spp. (*Lampetra fluvialitis* and *Lampetra planeri*), least brook lamprey (*Lampetra aepyptera*), Southern brook lamprey (*Ichtyomyzon gagei*), Northern brook lamprey (*Ichtyomyzon fossor*), (*Ichtyomyzon uyicuspis*) and Artic lamprey (*Lethenteron camtschaticum*). (B) Alignment with designed with references with accession numbers U11880.1 and NC001626.1 (*P. marinus* full mitogenome) and FR669669.2, FR669670.2, FR669671.2 and AJ937946.1 (*L. planeri* and *L. fluviatilis*).

**Supporting Information Table S1**BLAST statistics for individual O1_CR5_H21
**Supporting Information Table S2**BLAST statistics for individual O2_CR5_J21
**Supporting Information Table S3**BLAST statistics for individual O3_CR5_L21
**Supporting Information Table S4**BLAST statistics for individual SP3_LF_J23
**Supporting Information Table S5**BLAST statistics for individual SP4_LF_L23
**Supporting Information Table S6**BLAST statistics for individual Sp5_LF_N23
**Supporting Information Table S7**BLAST statistics for the forward primer utilized on the qPCR assay
**Supporting Information Table S8**BLAST statistics for the reverse primer utilized on the qPCR assay
**Supporting Information Table S9**BLAST statistics for the probe utilized on the qPCR assay
