# Supplementary material for: A noninvasive eDNA tool for detecting sea lamprey larvae in river sediments: Analytical validation and field testing in a low‐abundance ecosystem
Source: J Fish Biol. 2022 Apr 19;100(6):1455–63. doi: 10.1111/jfb.15056 (PMC9322552; doi:10.1111/jfb.15056)
Supplement: Supplementary file 1 — Supporting Information Figure S1Phylogenetic relationship based on the ATP8–ATP6 region. The quantitative PCR assay relied on primers designed to amplify only sea lamprey DNA. (A) Unrooted phylogenetic tree constructed with the neighbour‐joining method (Saitou & Nei, 1987) and utilizing 100 cyclostome sequences retrieved from the National Center for Biotechnology Information. Species utilized were the sea lamprey (Petromyzon marinus), Lampetra spp. (Lampetra fluvialitis and Lampetra planeri), least brook lamprey (Lampetra aepyptera), Southern brook lamprey (Ichtyomyzon gagei), Northern brook lamprey (Ichtyomyzon fossor), (Ichtyomyzon uyicuspis) and Artic lamprey (Lethenteron camtschaticum). (B) Alignment with designed with references with accession numbers U11880.1 and NC001626.1 (P. marinus full mitogenome) and FR669669.2, FR669670.2, FR669671.2 and AJ937946.1 (L. planeri and L. fluviatilis). [file JFB-100-1455-s001.pdf]

(A)

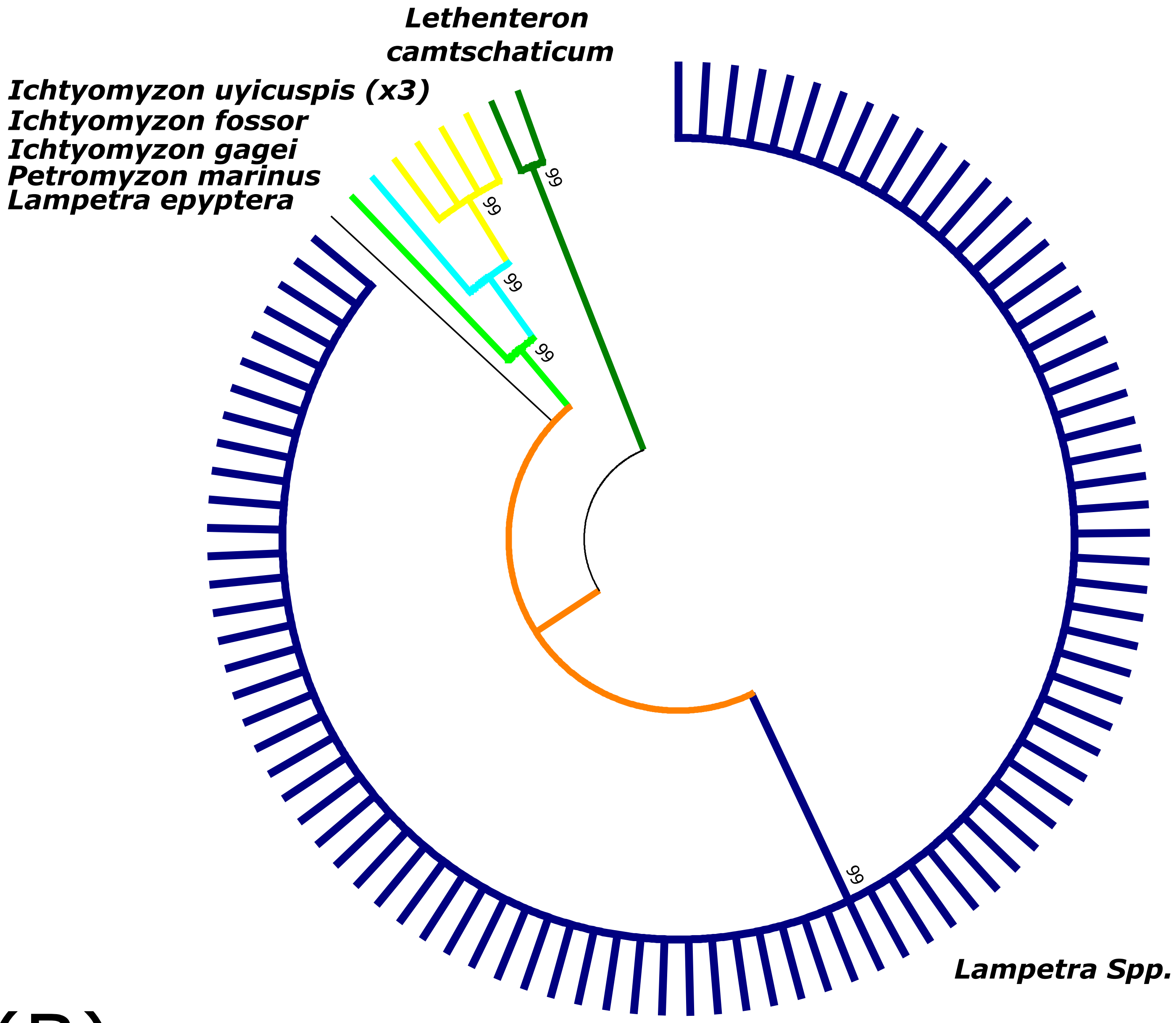
Lethenteron camtschaticum
Ichtyomyzon uyicuspis (x3)
Ichtyomyzon fossor
Ichtyomyzon gagei
Petromyzon marinus
Lampetra epyptera
99
99
99
99
99
Lampetra Spp.

(B)

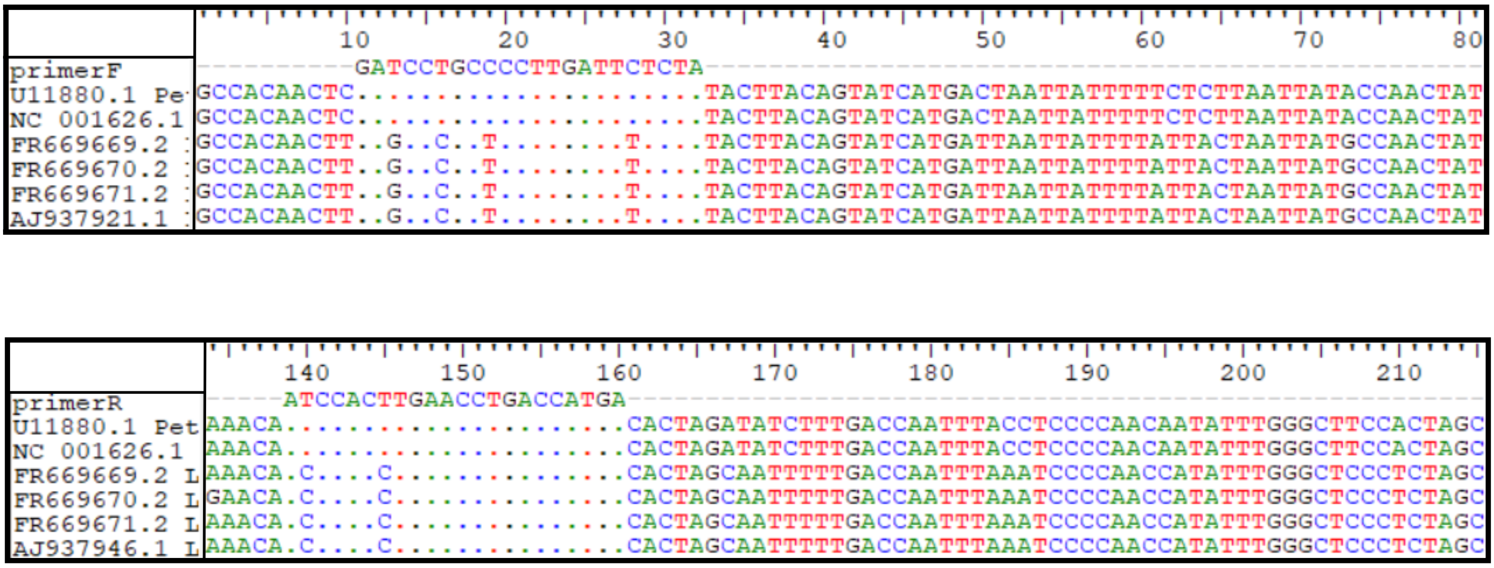
10 20 30 40 50 60 70 80
primerF ----------GATCCTGCCCCTTGATTCTCTA------------------------------------------------
U11880.1 Pe GCCACAACTC......................TACTTACAGTATCATGACTAATTATTTTTCTCTTAATTATACCAACTAT
NC 001626.1 GCCACAACTC......................TACTTACAGTATCATGACTAATTATTTTTCTCTTAATTATACCAACTAT
FR669669.2 GCCACAACTT..G..C..T........T....TACTTACAGTATCATGATTAATTATTTTATTACTAATTATGCCAACTAT
FR669670.2 GCCACAACTT..G..C..T........T....TACTTACAGTATCATGATTAATTATTTTATTACTAATTATGCCAACTAT
FR669671.2 GCCACAACTT..G..C..T........T....TACTTACAGTATCATGATTAATTATTTTATTACTAATTATGCCAACTAT
AJ937921.1 GCCACAACTT..G..C..T........T....TACTTACAGTATCATGATTAATTATTTTATTACTAATTATGCCAACTAT
140 150 160 170 180 190 200 210
primerR -----ATCCACTTGAACCTGACCATGA-------------------------------------------------------
U11880.1 Pet AAACA......................CACTAGATATCTTTGACCAATTTACCTCCCCAACAATATTTGGGCTTCCACTAGC
NC 001626.1 AAACA......................CACTAGATATCTTTGACCAATTTACCTCCCCAACAATATTTGGGCTTCCACTAGC
FR669669.2 L AAACA.C....C...............CACTAGCAATTTTTGACCAATTTAAATCCCCAACCATATTTGGGCTCCCTCTAGC
FR669670.2 L GAACA.C....C...............CACTAGCAATTTTTGACCAATTTAAATCCCCAACCATATTTGGGCTCCCTCTAGC
FR669671.2 L AAACA.C....C...............CACTAGCAATTTTTGACCAATTTAAATCCCCAACCATATTTGGGCTCCCTCTAGC
AJ937946.1 L AAACA.C....C...............CACTAGCAATTTTTGACCAATTTAAATCCCCAACCATATTTGGGCTCCCTCTAGC
