# Supplementary material for: A noninvasive eDNA tool for detecting sea lamprey larvae in river sediments: Analytical validation and field testing in a low‐abundance ecosystem
Source: J Fish Biol. 2022 Apr 19;100(6):1455–63. doi: 10.1111/jfb.15056 (PMC9322552; doi:10.1111/jfb.15056)
Supplement: Supplementary file 2 — Supporting Information Table S1BLAST statistics for individual O1_CR5_H21 Supporting Information Table S2BLAST statistics for individual O2_CR5_J21 Supporting Information Table S3BLAST statistics for individual O3_CR5_L21 Supporting Information Table S4BLAST statistics for individual SP3_LF_J23 Supporting Information Table S5BLAST statistics for individual SP4_LF_L23 Supporting Information Table S6BLAST statistics for individual Sp5_LF_N23 Supporting Information Table S7BLAST statistics for the forward primer utilized on the qPCR assay Supporting Information Table S8BLAST statistics for the reverse primer utilized on the qPCR assay Supporting Information Table S9BLAST statistics for the probe utilized on the qPCR assay [file JFB-100-1455-s002.docx]

**Supplementary tables**

Table S1 – BLAST statistics for individual O1_CR5_H21

| Description | Reference | % Identity | *e-value* | bit score |
| --- | --- | --- | --- | --- |
| Lampetra fluviatilis complete mitochondrial genome | EU596096.1 | 100 | 0 | 769 |
| Lampetra fluviatilis haplotype Lfl04 NADH dehydrogenase subunit 6 gene, partial cds; noncoding region I, complete sequence; tRNA-Thr and tRNA-Glu genes, complete sequence; and noncoding region II, partial sequence; mitochondrial | EU596094.1 | 100 | 0 | 769 |
| Lampetra fluviatilis haplotype Lfl85 NADH dehydrogenase subunit 6 gene, partial cds; noncoding region I, complete sequence; tRNA-Thr and tRNA-Glu genes, complete sequence; and noncoding region II, partial sequence; mitochondrial | KR558640.1 | 99.76 | 0 | 763 |
| Lampetra fluviatilis voucher DB_124 NADH dehydrogenase subunit 6 (ND6) gene, partial cds; and control region, partial sequence; mitochondrial | GQ340535.1 | 99.76 | 0 | 763 |
| Lampetra fluviatilis voucher DB_17 NADH dehydrogenase subunit 6 (ND6) gene, partial cds; and control region, partial sequence; mitochondrial | EU596145.1 | 99.76 | 0 | 763 |
| Lampetra fluviatilis voucher DB_26 NADH dehydrogenase subunit 6 (ND6) gene, partial cds; and control region, partial sequence; mitochondrial | EU596143.1 | 99.76 | 0 | 763 |
| Lampetra fluviatilis voucher DB_38 NADH dehydrogenase subunit 6 (ND6) gene, partial cds; and control region, partial sequence; mitochondrial | EU596132.1 | 99.76 | 0 | 763 |
| Lampetra fluviatilis voucher LFLUST23 control region, partial sequence; mitochondrial | EU595965.1 | 99.76 | 0 | 763 |
| Lampetra fluviatilis voucher LPLAGI17 control region, partial sequence; mitochondrial | EU404082.1 | 99.76 | 0 | 763 |

Table S2 – BLAST statistics for individual O2_CR5_J21

| Description | Reference | % Identity | *e-value* | bit score |
| --- | --- | --- | --- | --- |
| Petromyzon marinus isolate A10LampFor NADH dehydrogenase subunit 6 gene, partial cds; control region, complete sequence; and tRNA-Thr gene, partial sequence; mitochondrial | JX484170.1 | 100 | 0 | 773 |
| Petromyzon marinus voucher PMT2 D-loop, partial sequence; mitochondrial | EF565737.1 | 100 | 0 | 773 |
| Petromyzon marinus voucher PMRH53 D-loop, partial sequence; mitochondrial | EF565716.1 | 100 | 0 | 773 |
| Petromyzon marinus voucher PMMG1 D-loop, partial sequence; mitochondrial | EF565709.1 | 100 | 0 | 773 |
| Petromyzon marinus voucher PMLI24 D-loop, partial sequence; mitochondrial | EF565694.1 | 100 | 0 | 773 |
| Petromyzon marinus voucher PMLI19 D-loop, partial sequence; mitochondrial | EF565689.1 | 100 | 0 | 773 |
| Petromyzon marinus voucher PMD3 D-loop, partial sequence; mitochondrial | EF565679.1 | 100 | 0 | 773 |
| Petromyzon marinus voucher PMLI17 D-loop, partial sequence; mitochondrial | EF565493.1 | 100 | 0 | 773 |
| Petromyzon marinus isolate M4LampFor NADH dehydrogenase subunit 6 gene, partial cds; control region, complete sequence; and tRNA-Thr gene, partial sequence; mitochondrial | JX484234.1 | 99.761 | 0 | 767 |

Table S3 – BLAST statistics for individual O3_CR5_L21

| Description | Reference | % Identity | *e-value* | bit score |
| --- | --- | --- | --- | --- |
| Petromyzon marinus isolate A10LampFor NADH dehydrogenase subunit 6 gene, partial cds; control region, complete sequence; and tRNA-Thr gene, partial sequence; mitochondrial | JX484170.1 | 100 | 0 | 773 |
| Petromyzon marinus voucher PMT2 D-loop, partial sequence; mitochondrial | EF565737.1 | 100 | 0 | 773 |
| Petromyzon marinus voucher PMRH53 D-loop, partial sequence; mitochondrial | EF565716.1 | 100 | 0 | 773 |
| Petromyzon marinus voucher PMMG1 D-loop, partial sequence; mitochondrial | EF565709.1 | 100 | 0 | 773 |
| Petromyzon marinus voucher PMLI24 D-loop, partial sequence; mitochondrial | EF565694.1 | 100 | 0 | 773 |
| Petromyzon marinus voucher PMLI19 D-loop, partial sequence; mitochondrial | EF565689.1 | 100 | 0 | 773 |
| Petromyzon marinus voucher PMD3 D-loop, partial sequence; mitochondrial | EF565679.1 | 100 | 0 | 773 |
| Petromyzon marinus voucher PMLI17 D-loop, partial sequence; mitochondrial | EF565493.1 | 100 | 0 | 773 |
| Petromyzon marinus isolate M4LampFor NADH dehydrogenase subunit 6 gene, partial cds; control region, complete sequence; and tRNA-Thr gene, partial sequence; mitochondrial | JX484234.1 | 99.761 | 0 | 767 |

Table S4 – BLAST statistics for individual SP3_LF_J23

| Description | Reference | % Identity | *e-value* | bit score |
| --- | --- | --- | --- | --- |
| Petromyzon marinus isolate A10LampFor NADH dehydrogenase subunit 6 gene, partial cds; control region, complete sequence; and tRNA-Thr gene, partial sequence; mitochondrial | JX484170.1 | 100 | 0 | 773 |
| Petromyzon marinus voucher PMT2 D-loop, partial sequence; mitochondrial | EF565737.1 | 100 | 0 | 773 |
| Petromyzon marinus voucher PMRH53 D-loop, partial sequence; mitochondrial | EF565716.1 | 100 | 0 | 773 |
| Petromyzon marinus voucher PMMG1 D-loop, partial sequence; mitochondrial | EF565709.1 | 100 | 0 | 773 |
| Petromyzon marinus voucher PMLI24 D-loop, partial sequence; mitochondrial | EF565694.1 | 100 | 0 | 773 |
| Petromyzon marinus voucher PMLI19 D-loop, partial sequence; mitochondrial | EF565689.1 | 100 | 0 | 773 |
| Petromyzon marinus voucher PMD3 D-loop, partial sequence; mitochondrial | EF565679.1 | 100 | 0 | 773 |
| Petromyzon marinus voucher PMLI17 D-loop, partial sequence; mitochondrial | EF565493.1 | 100 | 0 | 773 |
| Petromyzon marinus isolate M4LampFor NADH dehydrogenase subunit 6 gene, partial cds; control region, complete sequence; and tRNA-Thr gene, partial sequence; mitochondrial | JX484234.1 | 99.761 | 0 | 767 |

Table S5 – BLAST statistics for individual SP4_LF_L23

| Description | Reference | % Identity | *e-value* | bit score |
| --- | --- | --- | --- | --- |
| Petromyzon marinus isolate A10LampFor NADH dehydrogenase subunit 6 gene, partial cds; control region, complete sequence; and tRNA-Thr gene, partial sequence; mitochondrial | JX484170.1 | 100 | 0 | 773 |
| Petromyzon marinus voucher PMT2 D-loop, partial sequence; mitochondrial | EF565737.1 | 100 | 0 | 773 |
| Petromyzon marinus voucher PMRH53 D-loop, partial sequence; mitochondrial | EF565716.1 | 100 | 0 | 773 |
| Petromyzon marinus voucher PMMG1 D-loop, partial sequence; mitochondrial | EF565709.1 | 100 | 0 | 773 |
| Petromyzon marinus voucher PMLI24 D-loop, partial sequence; mitochondrial | EF565694.1 | 100 | 0 | 773 |
| Petromyzon marinus voucher PMLI19 D-loop, partial sequence; mitochondrial | EF565689.1 | 100 | 0 | 773 |
| Petromyzon marinus voucher PMD3 D-loop, partial sequence; mitochondrial | EF565679.1 | 100 | 0 | 773 |
| Petromyzon marinus voucher PMLI17 D-loop, partial sequence; mitochondrial | EF565493.1 | 100 | 0 | 773 |
| Petromyzon marinus isolate M4LampFor NADH dehydrogenase subunit 6 gene, partial cds; control region, complete sequence; and tRNA-Thr gene, partial sequence; mitochondrial | JX484234.1 | 99.761 | 0 | 767 |

Table S6 – BLAST statistics for individual Sp5_LF_N23

| Description | Reference | % Identity | *e-value* | bit score |
| --- | --- | --- | --- | --- |
| Petromyzon marinus isolate B7LampFor NADH dehydrogenase subunit 6 gene, partial cds; control region, complete sequence; and tRNA-Thr gene, partial sequence; mitochondrial | JX484187.1 | 100 | 0 | 771 |
| Petromyzon marinus voucher PMRH55 D-loop, partial sequence; mitochondrial | EF565718.1 | 100 | 0 | 771 |
| Petromyzon marinus voucher PMMG11 D-loop, partial sequence; mitochondrial | EF565706.1 | 100 | 0 | 771 |
| Petromyzon marinus voucher PMD5 D-loop, partial sequence; mitochondrial | EF565680.1 | 100 | 0 | 771 |
| Petromyzon marinus voucher PMMG29 D-loop, partial sequence; mitochondrial | EF565470.1 | 100 | 0 | 771 |
| Petromyzon marinus isolate A10LampFor NADH dehydrogenase subunit 6 gene, partial cds; control region, complete sequence; and tRNA-Thr gene, partial sequence; mitochondrial | JX484170.1 | 99.761 | 0 | 765 |
| Petromyzon marinus voucher PMT2 D-loop, partial sequence; mitochondrial | EF565737.1 | 99.761 | 0 | 765 |
| Petromyzon marinus voucher PMRH53 D-loop, partial sequence; mitochondrial | EF565716.1 | 99.761 | 0 | 765 |
| Petromyzon marinus voucher PMMG1 D-loop, partial sequence; mitochondrial | EF565709.1 | 99.761 | 0 | 765 |

Table S7 – BLAST statistics for the forward primer utilized on the qPCR assay

| Description | Reference | % Identity | *e-value* | max score |
| --- | --- | --- | --- | --- |
| Petromyzon marinus mitochondrion, complete genome | [U11880.1](https://www.ncbi.nlm.nih.gov/nucleotide/U11880.1?report=genbank&log$=nucltop&blast_rank=2&RID=NM9U7R1Z014) | 100 | 0.034 | 44.1 |
| Novaculichthys taeniourus isolate nthi629 ATPase subunit 8 and ATPase subunit 6 genes, complete cds; mitochondrial | [DQ111547.1](https://www.ncbi.nlm.nih.gov/nucleotide/DQ111547.1?report=genbank&log$=nucltop&blast_rank=3&RID=NM9U7R1Z014) | 100 | 0.13 | 42.1 |
| Novaculichthys taeniourus isolate NTHI568 ATPase subunit 8 and ATPase subunit 6 genes, complete cds; mitochondrial | [DQ111546.1](https://www.ncbi.nlm.nih.gov/nucleotide/DQ111546.1?report=genbank&log$=nucltop&blast_rank=4&RID=NM9U7R1Z014) | 100 | 0.13 | 42.1 |
| Novaculichthys taeniourus isolate NTHI673 ATPase subunit 8 and ATPase subunit 6 genes, complete cds; mitochondrial | [DQ111545.1](https://www.ncbi.nlm.nih.gov/nucleotide/DQ111545.1?report=genbank&log$=nucltop&blast_rank=5&RID=NM9U7R1Z014) | 100 | 0.13 | 42.1 |
| Novaculichthys taeniourus isolate NTHI628 ATPase subunit 8 and ATPase subunit 6 genes, complete cds; mitochondrial | [DQ111544.1](https://www.ncbi.nlm.nih.gov/nucleotide/DQ111544.1?report=genbank&log$=nucltop&blast_rank=6&RID=NM9U7R1Z014) | 100 | 0.13 | 42.1 |
| Novaculichthys taeniourus isolate NTM177 ATPase subunit 8 and ATPase subunit 6 genes, complete cds; mitochondrial | [DQ111541.1](https://www.ncbi.nlm.nih.gov/nucleotide/DQ111541.1?report=genbank&log$=nucltop&blast_rank=7&RID=NM9U7R1Z014) | 100 | 0.13 | 42.1 |
| Novaculichthys taeniourus isolate epb2 ATPase subunit 8 and ATPase subunit 6 genes, complete cds; mitochondrial | [DQ111539.1](https://www.ncbi.nlm.nih.gov/nucleotide/DQ111539.1?report=genbank&log$=nucltop&blast_rank=8&RID=NM9U7R1Z014) | 100 | 0.13 | 42.1 |
| Novaculichthys taeniourus isolate NTX299 ATPase subunit 8 and ATPase subunit 6 genes, complete cds; mitochondrial | [DQ111535.1](https://www.ncbi.nlm.nih.gov/nucleotide/DQ111535.1?report=genbank&log$=nucltop&blast_rank=9&RID=NM9U7R1Z014) | 100 | 0.13 | 42.1 |

Table S8 – BLAST statistics for the reverse primer utilized on the qPCR assay

| Description | Reference | % Identity | *e-value* | max score |
| --- | --- | --- | --- | --- |
| Petromyzon marinus mitochondrion, complete genome | [U11880.1](https://www.ncbi.nlm.nih.gov/nucleotide/U11880.1?report=genbank&log$=nucltop&blast_rank=2&RID=NMA86KCR01R) | 100 | 0.034 | 44.1 |
| Pipistrellus pipistrellus genome assembly, chromosome: 11 | [LR862367.1](https://www.ncbi.nlm.nih.gov/nucleotide/LR862367.1?report=genbank&log$=nucltop&blast_rank=3&RID=NMA86KCR01R) | 100 | 0.53 | 40.1 |
| PREDICTED: Mirounga leonina guanylate cyclase 1 soluble subunit beta 1 (GUCY1B1), transcript variant X3, mRNA | [XM_035022845.1](https://www.ncbi.nlm.nih.gov/nucleotide/XM_035022845.1?report=genbank&log$=nucltop&blast_rank=4&RID=NMA86KCR01R) | 100 | 2.1 | 38.2 |
| PREDICTED: Mirounga leonina guanylate cyclase 1 soluble subunit beta 1 (GUCY1B1), transcript variant X2, mRNA | [XM_035022844.1](https://www.ncbi.nlm.nih.gov/nucleotide/XM_035022844.1?report=genbank&log$=nucltop&blast_rank=5&RID=NMA86KCR01R) | 100 | 2.1 | 38.2 |
| PREDICTED: Mirounga leonina guanylate cyclase 1 soluble subunit beta 1 (GUCY1B1), transcript variant X1, mRNA | [XM_035022843.1](https://www.ncbi.nlm.nih.gov/nucleotide/XM_035022843.1?report=genbank&log$=nucltop&blast_rank=6&RID=NMA86KCR01R) | 100 | 2.1 | 38.2 |
| PREDICTED: Ailuropoda melanoleuca guanylate cyclase 1 soluble subunit beta 1 (GUCY1B1), transcript variant X4, mRNA | [XM_034661477.1](https://www.ncbi.nlm.nih.gov/nucleotide/XM_034661477.1?report=genbank&log$=nucltop&blast_rank=7&RID=NMA86KCR01R) | 100 | 2.1 | 38.2 |
| PREDICTED: Ailuropoda melanoleuca guanylate cyclase 1 soluble subunit beta 1 (GUCY1B1), transcript variant X3, mRNA | [XM_019804964.2](https://www.ncbi.nlm.nih.gov/nucleotide/XM_019804964.2?report=genbank&log$=nucltop&blast_rank=8&RID=NMA86KCR01R) | 100 | 2.1 | 38.2 |
| PREDICTED: Ailuropoda melanoleuca guanylate cyclase 1 soluble subunit beta 1 (GUCY1B1), transcript variant X2, mRNA | [XM_002924331.4](https://www.ncbi.nlm.nih.gov/nucleotide/XM_002924331.4?report=genbank&log$=nucltop&blast_rank=9&RID=NMA86KCR01R) | 100 | 2.1 | 38.2 |

Table S9 – BLAST statistics for the probe utilized on the qPCR assay

| Description | Reference | % Identity | *e-value* | max score |
| --- | --- | --- | --- | --- |
| Petromyzon marinus mitochondrion, complete genome | [U11880.1](https://www.ncbi.nlm.nih.gov/nucleotide/U11880.1?report=genbank&log$=nucltop&blast_rank=2&RID=NM9NDUX5014) | 100 | 0.034 | 44.1 |
| Aspergillus sojae strain SMF134 chromosome 1 | [CP035530.1](https://www.ncbi.nlm.nih.gov/nucleotide/CP035530.1?report=genbank&log$=nucltop&blast_rank=3&RID=NM9NDUX5014) | 100 | 0.53 | 40.1 |
| Aspergillus flavus strain Afla-Guard chromosome 1 | [CP051067.1](https://www.ncbi.nlm.nih.gov/nucleotide/CP051067.1?report=genbank&log$=nucltop&blast_rank=4&RID=NM9NDUX5014) | 100 | 0.53 | 40.1 |
| Synchytrium microbalum uncharacterized protein (SmJEL517_g04269), partial mRNA | [XM_031170197.1](https://www.ncbi.nlm.nih.gov/nucleotide/XM_031170197.1?report=genbank&log$=nucltop&blast_rank=5&RID=NM9NDUX5014) | 100 | 2.1 | 38.2 |
| Pan troglodytes BAC clone CH251-165C4 from chromosome 6, complete sequence | [AC187382.3](https://www.ncbi.nlm.nih.gov/nucleotide/AC187382.3?report=genbank&log$=nucltop&blast_rank=6&RID=NM9NDUX5014) | 100 | 2.1 | 38.2 |
| Homo sapiens chromosome 5 clone RPCI-1_220J2, complete sequence | [AC009191.5](https://www.ncbi.nlm.nih.gov/nucleotide/AC009191.5?report=genbank&log$=nucltop&blast_rank=7&RID=NM9NDUX5014) | 100 | 2.1 | 38.2 |
| Rhinatrema bivittatum genome assembly, chromosome: 17 | [LR584403.1](https://www.ncbi.nlm.nih.gov/nucleotide/LR584403.1?report=genbank&log$=nucltop&blast_rank=8&RID=NM9NDUX5014) | 100 | 2.1 | 38.2 |
| Pan troglodytes BAC clone CH251-427H8 from chromosome 6, complete sequence | [AC183951.3](https://www.ncbi.nlm.nih.gov/nucleotide/AC183951.3?report=genbank&log$=nucltop&blast_rank=9&RID=NM9NDUX5014) | 100 | 2.1 | 38.2 |
| Pan troglodytes BAC clone CH251-545A14 from chromosome 6, complete sequence | [AC183671.2](https://www.ncbi.nlm.nih.gov/nucleotide/AC183671.2?report=genbank&log$=nucltop&blast_rank=10&RID=NM9NDUX5014) | 100 | 2.1 | 38.2 |
